# Test–retest reliability and construct validity of the Aspects of Wheelchair Mobility Test as a measure of the mobility of wheelchair users

**DOI:** 10.4102/ajod.v6i0.331

**Published:** 2017-09-08

**Authors:** Karen L. Rispin, Kara Huff, Joy Wee

**Affiliations:** 1 Department of Biology, LeTourneau University, United States; 2 School of Rehabilitation Therapy, LeTourneau University, United States; 3 School of Rehabilitation Therapy, Queen’s University, Canada

## Abstract

**Background:**

The Aspects of Wheelchair Mobility Test (AWMT) was developed for use in a repeated measures format to provide comparative effectiveness data on mobility facilitated by different wheelchair types. It has been used in preliminary studies to compare the mobility of wheelchairs designed for low-resource areas and is intended to be simple and flexible enough so as to be used in low-technology settings. However, to reliably compare the impact of different types of wheelchairs on the mobility of users, a measure must first be a reliable and valid measure of mobility.

**Methods:**

This study investigated the test–retest reliability and concurrent validity for the AWMT 2.0 as a measure of mobility. For reliability testing, participants in a low-resource setting completed the tests twice in their own wheelchairs at least one week apart. For concurrent validity, participants also completed the Wheelchair Skills Test Questionnaire (WST-Q), a related but not identical validated assessment tool.

**Results:**

Concurrent validity was indicated by a significant positive correlation with an r value of 0.7 between the WST-Q capacity score and the AWMT 2.0 score. Test–retest reliability was confirmed by an intraclass correlation coefficient greater than 0.7 between the two trials.

**Conclusion:**

Results support the preliminary reliability and validity of the AWMT 2.0, supporting its effectiveness in comparing the mobility provided by different wheelchair types. This information can be used to enable effective use of limited funds for wheelchair selection at individual and organisational scales.

## Introduction

Comparative effectiveness studies on the mobility facilitated by wheelchairs designed for use in low-resource areas are lacking (Harniss, Samant Raja & Matter 2015; Matter et al. 2017; Pearlman et al. 2008). The Aspects of Wheelchair Mobility Test (AWMT) was developed as a physical performance measure to provide comparative effectiveness data on wheelchairs designed for low-resource settings (Rispin & Wee 2013, 2015). The AWMT, which is described in a companion paper in this journal, is intended to be used in a repeated measures format to assess the impact of wheelchair type on mobility in commonly encountered rolling environments (Rispin & Wee 2013, 2015).

In order to be useful as a measure of the impact on mobility of different wheelchair types, the AWMT must first be a valid and reliable measure of a wheelchair user’s mobility (Dijkers et al. 2002; Jerosch-Herold 2005; Kottner et al. 2011). Results can then be used with confidence to enable effective use of limited funds (Dijkers, Murphy & Krellman 2012; Revicki et al. 2008; Speight & Barendse 2010). Test–retest reliability is the variation in measurements taken by a single instrument on the same item, under the same conditions, after an interval of time (Dijkers et al. 2002; Kottner et al. 2011). A measure is considered reliable when variation between the two iterations meets an acceptance criterion, often set at an intraclass correlation (ICC) value greater than or equal to 0.7 (Dijkers et al. 2002; Kottner et al. 2011). Concurrent validity confirms that an assessment measures its target construct as indicated by significant correlation with a similar but not identical validated outcomes measure (Dijkers et al. 2002).

As described in the accompanying article (Rispin, Hamm & Wee, 2017), the AWMT uses measured tracks on four rolling environments: rough, smooth, tight spaces and curbs. These are commonly encountered in low-resource settings and were thought likely to discriminate differences because of wheelchair design. Although exercise heart rate was monitored, it was not found as effective as distance travelled during the timed test in differentiating between wheelchair types in each rolling environment (Rispin & Wee 2013, 2015). In earlier studies using AWMT 1.0, the duration of tests differed. On rough and smooth tracks, 6-min durations similar to the 6-min timed walk test were used (Crapo et al. 2002; Rispin & Wee 2015). On curb and tight tracks, 3-min durations were used as these rolling environments were thought to be too difficult or awkward for 6-min tests (Rispin & Wee 2015). The AWMT 1.0 3-min duration enabled discrimination between wheelchair types in past studies on curb and tight tracks (Rispin & Wee 2015). However, the shorter time period may have reduced sensitivity to change (Kosak & Smith 2005). The purpose of this study was to investigate test–retest reliability and construct validity of the updated AWMT 2.0 as a measure of the mobility of wheelchair users. We hypothesised that test–retest reliability would be confirmed by ICC above 0.7. Construct validity would be confirmed by significant correlation with a related validated measure. We also hypothesised that the elimination of exercise heart rate and making all timed tests a uniform 4 min length would simplify the AWMT 2.0.

## Methods

### Study site

This study was conducted in partnership with an organisation providing rehabilitation at a school for students with disabilities in a low-resource area. In their daily routine as they move between dorms, classrooms and dining halls, wheelchair users regularly traverse paved and unpaved areas, as well as curbs and tight spaces.

### Participants

Participants were a convenience sample consisting of all wheelchair using secondary school students who chose to participate in the study. Participation in the study was voluntary, and participants could withdraw at any time or choose not to complete any task. The study was conducted using the English language. All participants had completed an English proficiency exam as part of the admissions process to the secondary school, and English was the language of schooling. However, most of the participants spoke several languages, and the language used in casual conversation was a patois of several local languages mixed with some English.

### Protocol

Measured tracks incorporating rough, smooth, curbs and tight spaces rolling environments were established and the length of each track was measured using a survey wheel. The 60 m rough track was on an earth and gravel road. The best approximation to a smooth surface found was the somewhat uneven cement floor of the school dining hall and a 40 m track was set up around the periphery of the room. The curb was a wooden raised area 1.5 m wide and 7 cm tall on an outdoor cement surface, and the 11 m curb track traversed the raised area twice each loop. If a participant was unable or chose not to ascend the curb, no distance was measured. For the tight spaces track, four chairs were set in a row 1 m apart on an indoor cement floor. The 12 m track was a figure eight around the middle two of the four chairs. There was no time penalty for displacing a chair, but participants had been asked to avoid the chairs and seemed to be trying to do so.

Using their own wheelchairs, wheelchair users participated twice with a one-week intervening period. Wheelchair settings were not altered and wheelchairs were not repaired between iterations. Participants were invited to roll at a comfortable pace for 4 min on each track. They were reminded that they were free to withdraw from any test or stop and rest if needed during the tests. Instructions were similar to those established for the long validated American Thoracic Association Timed Walk Test (Crapo et al. 2002; Graham et al. 2008). After completing each track, participants completed a visual analogue scale question on the ease or difficulty of rolling on that track. The question also included an opportunity to provide an explanatory comment. The question format is described in more detail in the accompanying paper and was like that used in the Lower Limb Function Questionnaire and the Wheelchair Components Questionnaire (Funk et al. 2017; Rispin et al. 2017). Although heart rate had been monitored using research-grade heart rate monitors in earlier AWMT 1.0 studies, this was not performed in the updated AWMT 2.0 because heart rate had not consistently differentiated between wheelchair types. However, non-exercise heart rate was recorded before testing was initiated. Participants rested quietly for 5 min, and at the end of that time their brachial pulse was taken for 30 s. Each subsequent test was started only when a participant’s heart rate had returned to his or her non-exercise heart rate and the participant indicated that he or she was ready to begin. Participants were pushed between tracks to avoid fatigue. A low discrepancy shifting pattern of rolling environments was used to avoid skewing of results by the order of testing. Each person used the same order of testing in each iteration, but the order of tracks varied between participants so the track that was completed first, second and so on differed among participants.

The Wheelchair Skills Test Questionnaire (WST-Q version 4.2) was chosen as a related but not identical construct for the purpose of concurrent validity testing (Mountain, Kirby & Smith 2004). The WST-Q is a questionnaire version of the Wheelchair Skills Test, a physical performance measure intended to assess a wheelchair user’s skill level and capacity. The WST-Q asks a wheelchair user to assess their capacity and frequency of use of 32 skills and to indicate training goals. For the capacity score, participants rate their capacity on a scale of 0 to 2, with 0 being unable, 1 being able to complete, but not as well as the wheelchair user would like and 2 being able to do the skill safely and without difficulty. Administration of the WST-Q was done in a group setting in the school dining hall. Instructions were read aloud. While participants completed the WST-Q, researchers and facilitators circled the room to answer vocabulary questions because some of the terms in the WST-Q were unfamiliar to some participants.

### Analysis

The MiniTab statistical analysis program was used for data analysis. Data sets were tested for normality with the Anderson–Darling test. For concurrent validity, the mean distance travelled and the mean visual analogue score for both iterations for each participant were correlated with that of the participant’s WST-Q capacity score. For test–retest reliability, ICC was calculated for the two iterations for each participant’s total distance travelled on all tracks and mean visual analogue scale scores. IBM Statistical Package for the Social Science was used for ICC. Although we collected quantitative comments as is normal for the AWMT 2.0 and WST-Q, this study is focused on quantitative data which can be evaluated using ICC and correlation analysis.

### Ethical considerations

The study protocol was approved by the authors’ universities and partner organisations. Participants over 18 years of age provided informed written consent. Those under 18 years of age provided informed written assent and their guardians provided informed written consent.

## Results

### Participants

There were a total of 64 wheelchair users present at the secondary school. Of these, 50 users chose to participate (average age: 17.3 SD ± 1.75; gender: 27 male and 23 female). See Table 1 for information provided by participants on their wheelchair type and diagnoses. All participants completed the smooth track, 49 completed the tight track, 46 completed the rough track and 27 completed the curb track.

**TABLE 1a:** Types of wheelchairs as provided by the host organisation.

Number of participants	Type of wheelchairs
13	Chinese made folding transport
11	Motivation
7	Association of the Physically Disabled of Kenya
5	Wheelchair for Kids
3	Donated wheelchair of undesignated type
3	Hope Haven KidChair
3	Whirlwind
2	Quickie
1	Breezy Rubix
1	Invacare
1	Wheelchair Foundation

*Source*: Authors’ own work

**TABLE 1b:** Diagnosis as provided by the host organisation.

Number of participants	Diagnosis
17	Spinal conditions†
15	Limb deficiencies‡
11	Muscular dystrophy
5	Cerebral palsy
2	Polio

*Source*: Authors’ own work

† , This diagnosis includes 10 traumatic spinal injury, 6 spina bifida and 1 tuberculosis of the spine.

‡ , This diagnosis includes 11 osteogenesis imperfecta, 3 arthrogryposis and 1 bilateral amputation.

### Statistical results

The ICC result for each participant’s mean visual analogue score for all tracks for test and retest was 0.801 with a 95% confidence interval of 0.731–0.853. The ICC result for mean total distance travelled on all tracks for test and retest was 0.966 with a 95% confidence interval of 0.954–0.975. The total distance travelled correlated positively and significantly with the WST-Q capacity score with a Pearson’s correlation of 0.7 (*P* < 0.001). The mean visual analogue score response of participants also correlated positively and significantly with their WST-Q capacity score with a Pearson’s correlation of 0.49 (*P* < 0.001).

## Discussion

The purpose of this study was to investigate the reliability and validity of the AWMT 2.0 as a physical performance measure of mobility. Test–retest ICC results well above 0.7 confirm reliability for distance travelled and visual analogue score responses. Significant correlations with the WST-Q capacity scores confirm validity. The WST-Q is a validated physical performance measure. If the AWMT 2.0 is measuring physical performance aspects of mobility, one would expect significant positive correlation between the two measures. This was the case, and concurrent validity of the AWMT 2.0 was confirmed by positive and significant correlation between the total WST-Q capacity score and AWMT 2.0 distance travelled on all tracks. This was also the case for the correlation of AWMT 2.0 mean visual analogue score and WST-Q capacity score. In fact, these positive correlations emphasise the validity of both measures. One might ask why the AWMT 2.0 is needed if the WST-Q is a validated measure. The WST-Q is designed, as the name describes, to test the skill level of an individual wheelchair user. As such, the WST-Q is not primarily designed for comparative effectiveness studies on the impact on mobility of different wheelchair types. If a very strong wheelchair user can roll on rough ground, it is likely that he or she could do that in most wheelchair types, and the 0–2 categorical rating scale might not pick up a slower velocity or a greater difficulty in one wheelchair type as compared to another. Because of the categorical nature of WST-Q questionnaire data for each question, analysis of variance (ANOVA) could not be used to compare the impact of different wheelchair designs on capacity for each skill. If all 32 questions of the WST-Q were to be used, there would be greater discriminative power. However, this would be difficult to do in a repeated measures study because each participant would need to complete or attempt to complete many skills in each wheelchair. This would be very time-consuming and physically wearing. In contrast, because the data are continuous, ANOVA can be applied to AWMT data with rolling environments and wheelchair types acting as factors (see the accompanying paper).

The curb and rough tracks tests were included as part of the AWMT partly to prevent a ceiling effect; therefore, lack of completion on those tests is not unexpected. However, in earlier comparative studies, participants were selected for ability to self-propel without stress on rough surfaces (Rispin & Wee 2013, 2015). This was done with an attempt to enable more nearly complete data sets on all surfaces for greater statistical power for the ANOVA comparisons across tracks and wheelchair types. In this study, because we were not seeking to do a comparative effectiveness study and because wide variation is helpful in reliability and concurrent validity studies, all wheelchair users at the secondary school were invited to participate. All participants could propel on a smooth surface and most could propel on a rough surface and in tight spaces. Completion rates were somewhat lower on rough track and much lower on the curb track. Although all participants completed the smooth track, some rolled very slowly. Those who rolled very slowly even on the smooth track were most often those who did not normally propel themselves without help as they travelled around campus. Many had a friend, usually another student in the same courses, who assisted them as they rolled between classes, to the dorm and dining hall. In more developed areas, those who need regular assistance would likely have received a power wheelchair, but power wheelchairs are not yet broadly available in low-resource areas (Pearlman et al. 2009). Therefore, the mobility a manual wheelchair affords to a user or assistant team is also of interest. Work on the validation of a similar set of tests for wheelchair assistants pushing wheelchairs is also underway (Sasaki & Rispin 2016).

Our study population was not typical of all wheelchair users. Many wheelchair users are older people who have acquired disabilities. With a mean age of 17 years, participants in this study were younger, and there had been a strong selection process involved in their successful admittance into secondary school. Life for those with disabilities in low-resource areas is challenging (Borg, Lindström & Larsson 2011; Harniss et al. 2015). These teens and young adults were exceptional people with exceptional support networks. They had done well in primary school, passed the rigorous exam for admission to secondary school and were attending school. This required a support network for the payment of school fees, and to enable travel to and from boarding school. In an environment where power wheelchairs and devices to augment communications skills are not available, anyone who is unable to self-propel or has difficulty in writing or speaking is at a very great disadvantage. It is not surprising that our participants could write, speak and self-propel. However, this is likely not the case with the broader global population of wheelchair users.

In this study, unlike earlier studies, all tracks were of 4-min duration. On the rough and smooth tracks, the removal of 2 min seemed to reduce stress for the participants. On the curb and tight tracks, the addition of 1 min did not seem to add much stress for the participants. Because all tests were for a 4-min duration, distance travelled could be directly compared across tracks in future comparative studies. The modification of the AWMT 2.0 to eliminate the need of using research-grade heart rate monitors simplified the protocol in several ways. It removed the need to check batteries, fit and calibrate the monitors, download data and calculate mean exercise heart rate. This simpler protocol should increase the ease of use of the AWMT 2.0 in low-resource areas.

In large comparative effectiveness studies, the AWMT 2.0 can provide data on wheelchair types as is described in the accompanying article. Because the AWMT 2.0 is designed to assess the impact of different wheelchair types on the mobility of the same user, it could also be used to assess change across time. For example, the AWMT 2.0 could be used to document the impact on users’ mobility before and after rehabilitation treatments. In a clinical setting in which therapists work with one client at a time, AWMT 2.0 could be used to enable objective comparative input on wheelchair types for individual clients. Wheelchair users could have the option of trying out several wheelchairs by using the AWMT 2.0 to test the mobility provided to them by each wheelchair type. Results of the AWMT could be used as one component taken into account in the selection of a wheelchair type for a user. In many locations, letters of medical necessity, or some sort of equivalent, for provision are needed and objective evidence would reinforce the validity of a request (Greer, Brasure & Wilt 2012). If thresholds were selected for different rolling environments, AWMT 2.0 results could provide objective evidence of the need for a powered wheelchair. For example, a low distance travelled while rolling forward for 4 min on a rough surface track could indicate the need for a power chair for use outdoors.

### Study limitations and future work

There were some differences between the test and retest iterations. All tracks were completed at outdoor ambient temperatures, which varied over time. The rough surface track was on an unpaved road. Over the time period of testing, there were several rainy days. Although testing was delayed until the road had dried, road texture was different on a damp day after a rain than it was after several days of dry weather. The intervals between test and retest were not perfectly uniform because waiting on the weather sometimes delayed testing. We sought to minimise disruption to participants’ schedules; therefore, the time of day testing was conducted was not always the same for each participant in the initial and final iteration. Good test–retest reliability for distance travelled and participant response visual analogue scores indicates that even on slightly varying surfaces, differing times of day and temperatures, the AWMT 2.0 provided a reliable measurement of mobility.

Because the AWMT has been used with tracks set up on rough, smooth, curb and tight environments available on location, the protocol has a high innate variability. The smooth and tight environments could be set up identically at most locations. For the other two surfaces, the study would be generally repeatable if instead of locally available areas, standardised curb and rough surfaces units could be built. For example, for the rough surface, standardised modular rough surface could be built locally (Duvall et al. 2013; Sasaki & Rispin 2016). This is planned for an upcoming study in Kenya.

As described above, the population involved in this study was not representative of the global population of wheelchair users in low-resource settings. Therefore, further studies are also needed with other populations. The accompanying study on discriminatory validity had test durations of 3 and 6 min rather than the updated 4 min and included the use of heart rate monitors. Studies are also planned to confirm the discriminatory validity of the AWMT 2.0. Visual analogue scale format was chosen for the AWMT response question because it produced continuous data, which offers higher discriminatory power than categorical format scales such as the Likert scale (Philip 1990). However, the need to measure and record visual analogue scale results adds a time requirement that could be difficult to sustain in a clinical setting (Reips & Funke 2008). The visual analogue scale format is now becoming available in digital format (Reips & Funke 2008). Work is underway to provide AWMT 2.0 visual analogue scale questions in a small downloadable program. Globally, computers are often available in clinical settings. A digital version of the visual analogue scale questions would reduce the load on busy clinicians or researchers who wish to use the AWMT 2.0.

## Conclusion

These findings indicate that the AWMT 2.0 is a reliable and valid measure of a wheelchair user’s mobility. This confirms the soundness of comparative effectiveness studies conducted using the AWMT 2.0 for similar populations. As a clinical tool, the AWMT 2.0 could enable wheelchair fitting by allowing direct objective comparisons of the mobility provided by wheelchairs and configurations. In larger studies comparing cadres of wheelchairs of two or more types, the AWMT 2.0 can provide comparative effectiveness data to manufacturers, clinics and stakeholders. Because this study was conducted with a population of adolescent participants in one low-resource area, further studies are needed for other populations and in other locations.
